# The analysis of RCAS1 and DFF-45 expression in nasal polyps with respect to immune cells infiltration

**DOI:** 10.1186/1471-2172-7-4

**Published:** 2006-03-21

**Authors:** Magdalena Dutsch-Wicherek, Romana Tomaszewska, Pawel Strek, Lukasz Wicherek, Jacek Skladzien

**Affiliations:** 1 ENT Head and Neck Surgery Department, the Jagiellonian University, Krakow, Poland; 2 Pathomorphology Department, the Jagiellonian University, Krakow, Poland; 3 Gynecology and Infertility Department, the Jagiellonian University, Krakow, Poland

## Abstract

**Background:**

Nasal polyp constitutes a benign growth process in the nasal and sinus mucosa. RCAS1 (receptor-binding cancer antigen expressed on SiSo cells) is a protein expressed mainly by various human cancer cells. It is not only the marker of cancer process and its expression can also be observed in physiological processes. It is responsible for the regulation of immune cells activity. DFF45 (DNA fragmenting factor) has been described as a substrate for caspase-3. DFF45 seems to play an important role in the onset of apoptotic process by acting probably through the regulation of DNA fragmentation. The aim of the study was to evaluate the ability of nasal polyps to regulate the cytotoxic immune response and to determine their resistance to apoptosis.

**Results:**

The higher RCAS1 level was identified in lymphocytic nasal polyps, the medium one in eosinophilic while the lowest was identified in neutrophilic. DFF-45 expression was higher in eosinophilic than in neutrophilic and lymphocytic nasal polyps.

**Conclusion:**

The changes in DFF-45 level in nasal polyps might indicate a different resistance to apoptosis mediated by immune cells. The alterations in RCAS1 expression indicate that nasal polyps have the ability to regulate the cytotoxic immune response.

The breaking of resistance to immune mediated apoptosis in nasal polyps might have a new therapeutic impact.

## Background

Nasal polyp constitutes a benign growth process in the nasal and sinus mucosa which is mainly located in the middle meatus and never in the inferior meatus. The etiology of nasal polyps, which is a common clinical condition, is not well understood. Infections, allergy and immunological factors are considered.

Histopathologically polyps surface is covered by a ciliated pseudostratified epithelium and the subepithelial area is characterized by an eosinophilic inflammation in more than 80% of cases. The density of goblet cells in nasal polyps is much lower than in the normal nasal mucosa. The glands of nasal polyps are long, tubular, of varying shape, size and type, and their density is more than 10 times lower than in nasal mucosa [1-3].

Receptor-binding cancer antigen expressed on SiSo cells (RCAS1) is a protein which has been demonstrated in various human cancer cells responsible for tumour escape from host immunological surveillance. RCAS1 is not only the marker of cancer process, but its expression has also been observed in physiological conditions and the development of non-neoplastic tumours [4-11]. It has been demonstrated in the bone marrow, the endometrium, the decidua, the placenta, Waldeyer's ring and immune mediated diseases. RCAS1 seems to be responsible for the regulation of cytotoxic cells activity [10-18]. Within Waldeyer's ring RCAS1 has been expressed by reticular epithelial cells lining the tonsils. The reticular epithelium enables the communication between the antigen and the germinal center, which activates an immunological response [14]. This role of RCAS1 in the regulation of immune cells activity has also been confirmed in the creation of maternal immune tolerance during pregnancy. The expression of RCAS1 in the healthy endometrium depended on the hormonal cycle fluctuations and was related to the immune cytotoxic activity changes [16-18].

DNA fragmentation factor (DFF) is composed of a 40 kDa protein DFF40, with a nuclease activity, and a 45 kDa protein inhibitory subunit DFF45 [19]. Since DFF45 is able to interact with this nuclease as a molecular chaperone and ensures its correct folding it has been shown to be required for generating functional DFF40 nuclease [20]. It has been demonstrated that DFF-45-deficient thymocytes are more resistant to programmed cell death [21]. In esophageal and colon carcinoma cells characterized by an impairment of apoptosis, a decrease of DFF45 level has also been observed [22]. During apoptosis DFF45 was described as a substrate for caspase-3. DFF45 seems to play an important role in the onset of apoptotic process by acting probably through the regulation of DNA fragmentation [23].

The aim of the study was to evaluate the ability to regulate the cytotoxic immune response (RCAS1 expression) and to determine the resistance to apoptosis (DFF-45 expression) in nasal polyps.

## Results

Eosinophilic nasal polyps constituted almost 52.5% of examined samples, the infiltration of lymphocytes was predominant in 26.3% of nasal polyps and neutrophil concentration was observed in 21.2%.

### RCAS1 expression in nasal polyps

**Figure 1:**
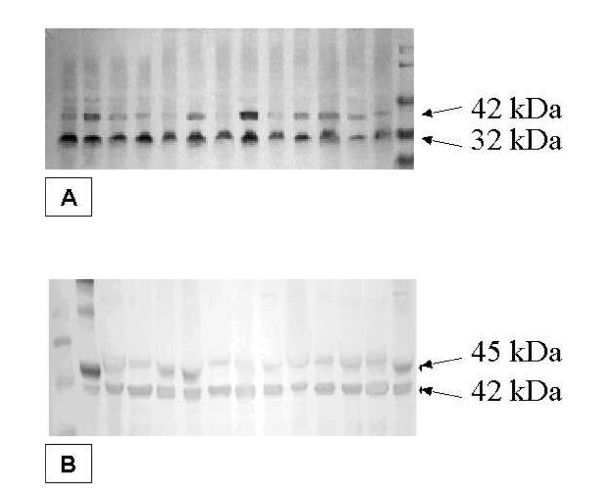
The results of RCAS1 (A), DFF45 (B) and beta-Actin expression in Western blot method. RCAS represented a 32 kDa band, DFF45 a 45 kDa band and beta-Actin a 42 kDa band.

**Figure 2:**
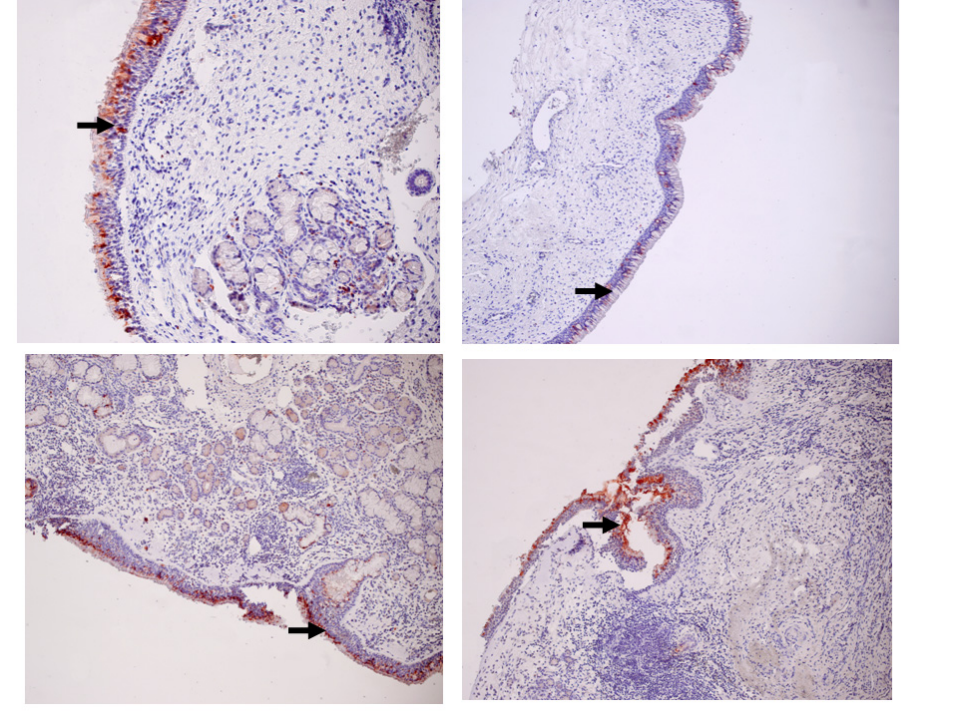
Nasal polyps. RCAS1 immunoreactivity in superficial layer of epithelium (vertical arrow).

**Figure 3:**
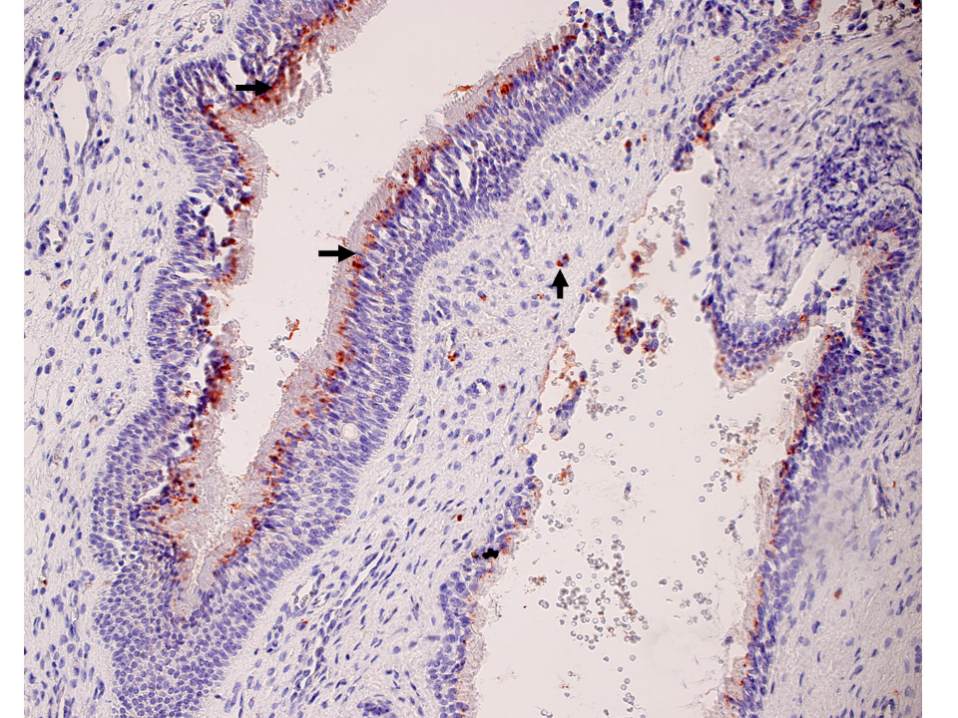
Nasal polyps. RCAS1 positive macrophages (vertical arrow) within polypoid stroma and RCAS1 immunoreactivity in superficial layer of epithelium (horizontal arrow).

RCAS1 was identified in all examined tissue samples as a 32 kDa band (Figure 1). The highest RCAS1 relative amount was identified in nasal polyps predominantly infiltrated by lymphocytes and it was statistically significantly higher than in eosinophilic nasal polyps (p = 0.01). The lowest RCAS1 expression level was observed in neutrophilic nasal polyps, and it was statistically significantly lower than in eosinophilic nasal polyps (p = 0.01) and statistically significantly lower than in lymphocytic nasal polyps (p < 0.001). The RCAS1 expression was confirmed by immunohistochemistry method in all examined tissue samples. A correlation of RCAS1 expression between Western blotting and immunohistochemistry was identified (R = 0.67, p < 0.001). RCAS1 immunoreactivity was found in superficial layer of pseudostratified ciliated epithelium covering the surface of nasal polyps (Figure 2). Moreover, RCAS1 expression was also identified in macrophages of polypoid stroma (Figure 3).

### DFF45 expression in nasal polyps

DFF45 expression was identified in all examined tissue samples as a 45 kDa band (Figure 1). DFF45 expression was statistically significantly higher in eosinophilic nasal polyps in comparison to lymphocytic nasal polyps (p < 0.0001) and significantly higher in eosinophilic nasal polyps than in neutrophilic (p < 0.001). DFF45 expression was at a comparable level in neutrophilic and lymphocytic nasal polyps.

The obtained results of RCAS1, DFF-45 and Beta-Actin control protein in examined tissue samples are presented in Table 1.

**Table 1:** The average relative amounts of RCAS1, DFF-45 and β-Actin in nasal polyps tissue samples with respect to the immune cells infiltration.

Nasal polyps divided according to immune cell concentration	Average relative amount of RCAS1 ± SD	Average relative amount of DFF-45 ± SD	Average relative amount of beta-Actin ± SD
Eosinophilic nasal polyps (n = 42)	1.3421 (± 0,6464)	0.6261 (± 0.1642)	0.9411 (± 0.2358)*
Lymphocyte infiltration (n = 21)	1.9345 (± 0.7121)	0.3603 (± 0.1157)	0.8722 (± 0.2526)*
Neutrophil infiltration (n = 17)	(± 0.2522)	(± 0.1409)	0.9308 (± 0.1883)*

• no differences statically significant, (n – number of patients)

The fact that the amount of beta-Actin in all groups of examined nasal polyps was found to be identical (Table 1) indicates that the loading of protein was equal in all samples examined and allows to perform a comparative study in RCAS1 expression between examined groups.

## Discussion

In the presented study statistically significant differences in RCAS1 and DFF45 expression in nasal polyps were identified according to the predominant immune cell infiltration type: eosinophils, lymphocytes and neutrophils.

The regulation of polypoid growth is biochemically determinated by various factors, like galectin-3, known of anti-apoptotic activity, which expression is markedly higher in nasal polyps than in nasal turbinate [27]. Apoptosis is defined as programmed cell death. It has been reported that apoptosis mediated through the interaction of Fas and Fas-L might participate in immune privilege of nasal polyps [28]. Fas/Fas-L interactions are an important mechanism in the functional organ development and in pathological changes such as cancer and immune mediated diseases through the participation in the regulation of the activity of immune cells [29]. The expression of Fas-L has been identified in both nasal polyps and nasal turbinate mucosa, but has been enhanced in nasal polyps. Moreover, Fas-L positive cells have been found in the down growing epithelium of nasal polyps and in the epithelial layer of cystically dilated glands [30]. Fas/Fas-L interaction results in the activation of the caspase cascade through FADD (Fas associated death domain) leading to the DFF-45 dissociation from DFF-40 and liberation of the DNA-ase responsible for the DNA fragmentation [31]. Ohshima compared FasL and RCAS1 expressions in Reed-Sternberg cells and trophoblast cells, and revealed that both proteins activate independently Fas-associated death domain which activates the caspase cascade and leads to the apoptosis of target cell. In this way the cells expressing RCAS1 and Fas-L are responsible for suppression of immune cytotoxic lymphocytes [13]. In both, Fas-L and RCAS1 pathways of apoptosis DFF-45/DFF-40 complex seems to be crucial. In the presented study RCAS1 immunohistochemically was identified only in the pseudostratified ciliated epithelium covering the surface of nasal polyps and this localization differs from the reported Fas-L localization in nasal polyps. RCAS1 positive macrophages were identified in polypoid tissue stroma. Until now RCAS1 positive macrophages have been identified in the bone marrow where they are responsible for the regulation of maturating erythroblasts [12]. Additionally RCAS1 positive macrophages have been found in ovarian endometriosis although they have not been present in the normal, eutopic endometrium [18]. In sum, it might be interpreted that RCAS1 positive macrophages might participate in the local immune dysfunction in nasal polyps.

The inflammatory infiltrate of nasal polyps consists of eosinophils, lymphocytes, mast cells, macrophage-like CD68+ cells [32]. Both eosinophils and lymphocytes are resistant to Fas-L/Fas mediated apoptosis [33]. The lymphocytes that infiltrate nasal polyps have been defined to be predominantly memory T cells in an activated status and produced a mixed Th1/Th2 cytokine pattern (IFN-gamma and IL-5) [34]. Cytokines, including IL-5, IFN-gamma, GM-CSF also participate in delaying of the death process in these cells, thus contributing to developing of tissue and blood eosinophilia [32,34,35]. The lymphocytes infiltrated nasal polyps in our study were characterized by the highest RCAS1 expression level and a statistically significantly lower DFF-45 expression than eosinophilic nasal polyps. Low DFF-45 level might indicate that the polyps infiltrated by lymphocytes are more resistant to immune mediated apoptosis than eosinophilic nasal polyps. Additionally, the expression of RCAS1, a factor participating in the suppression of cytotoxic activity, might help polypoid tissue to avoid immune cytotoxicity.

The etiology of neutrophilic nasal polyps is connected with bacterial inflammation. It has been postulated that superantigens derived mainly from *Staphylococcus aureus *might be potent activators of T cells and might therefore participate in the formation of nasal polyps [28]. Superantigens are able to activate the immune cytotoxic response without antigen presenting cells which results in the exposure of polypoid tissue to immune mediated apoptosis. In our study neutrophilic nasal polyps were characterized by a comparable level of DFF-45 as lymphocytic nasal polyps and lower than eosinophilic nasal polyps. We suspect that neutrophilic nasal polyps, similarly to lymphocytic nasal polyps, are more resistant to immune mediated apoptosis than eosinophilic nasal polyps, which might compensate the immune cytotoxicity. The observed decreased RCAS1 expression in neutrophilic nasal polyps might result from the immune cells infiltration pattern.

## Conclusion

The changes in DFF-45 level in nasal polyps regarding the type of immune cell infiltration might indicate a different resistance to apoptosis mediated by immune cells. The alterations in RCAS1 expression indicate that nasal polyps have the ability to regulate the cytotoxic immune response.

## Methods

### Clinical material

Tissue samples were derived from ENT Head and Neck Surgery Department of the Jagiellonian University during routine endonasal sinus surgery. In all cases patient's consent was obtained. The approval for the research program from the Ethical Committee of the Jagiellonian University in Krakow: KBET/379/13/2003 was also granted. We recruited 300 patients from the patients that undergone functional endoscopic sinus surgery between January 2003 and January 2005. From this group of patients 80 patients were randomly selected to our study. We obtained 80 tissue samples. All tissue samples were histopathologically verified. Pathological analysis using the classical hematoxylin and eosin staining techniques after fixation in formalin of the surgically removed material was performed in the Pathology Department of the Jagiellonian University by an experienced pathologist. The predominant immune cell infiltration in nasal polyps was determined. According to the predominant immune cell infiltration the tissue samples were divided into three main groups: nasal polyps predominantly infiltrated by eosinophils (eosinophilic nasal polyps)- 42 cases, nasal polyps predominantly infiltrated by lymphocytes (lymphocytic nasal polyps)- 21 cases and nasal polyps predominantly infiltrated by neutrophils (neutrophilic nasal polyps)-17 cases.

The clinical characteristics of subjects is presented in Table 2.

**Table 2:** The characteristics of subjects.

Variable	Mean age	BMI (body mass index)	Sex: Male Female	Surgical procedure
Eosinophilic infiltration (n = 42)	42.67 (± 14.09)	25.57 (± 3.38)	17 (40%) 25 (60%)	polypectomy 100% ethmoidectomy 93% sphenoidectomy 10% maxillectomy 30.7%
Lymphocytes infiltration (n = 21)	42.62 (± 17.09)	26.25 (± 3.24)	7 (33.3%) 14 (66.6%)	polypectomy 100% ethmoidectomy 94% sphenoidectomy 13% maxillectomy 30%
Neutrophils infiltration (n = 17)	43.6 (± 13.50)	26.14 (± 1.9)	6 (33%) 12 (67%)	polypectomy 100% maxillectomy 33%

### Western blotting

The Western blot analysis was necessary to elucidate the doubts concerning the specifity of anti-RCAS1 monoclonal antibodies. Engelsberg informs that anti-RCAS1 monoclonal antibodies recognize not only RCAS1 but also Thomsen-Friedenreich antigen (TF antigen) and Tn antigen in immunohistochemistry method [24]. In general TF (Galβ1-3GalNAc) and Tn (O-linked glycan structures like terminal GalNAc) antigens are thought to be linked to cells adhesion, invasion and metastases of cancer cells. The analysis performed by Engelsberg of recombinant RCAS1-GST fusion protein confirmed RCAS1 molecular weight described earlier by Tsuchija as a 32 kDa band in the Western blot method [24,25]. Tsuchija performed the analysis of molecular cloning EBAG9/RCAS1 gene. TF antigen is recognized by the jackfruit lectin, jacalin and molecular masses were established at: 230, 188, 168, 120 and 58 kDa levels [26]. Then we were sure that we recognized RCAS1 identifying its molecular weight at 32 kDa in the Western blot method.

#### Preparation of tissue extracts

Tissue samples (app. 0.5 × 0.5 × 0.5 cm) were obtained from the nasal polyps present within the middle nasal meatus collected during routine endonasal sinus surgery and were immediately frozen. The specimens were mixed with complete proteinase inhibitor cocktail (Roche, Germany) and homogenized on ice-bath in glass-glass Potter-Elvejhem homogenizer. The resulting suspensions were mixed with an equal volume of SDS sample lysis buffer (4% SDS, 20% glycerol, 125 mM Tris-HCl pH 6.8) and boiled in water bath for 5 minutes. The chilled samples were then centrifuged at 16,000 g at room temperature for 15 minutes. The collected supernatants were used for further analysis.

#### Western blotting

The total protein content in the obtained supernatants was measured using BCA assay kit and different sample volumes (usually in the range of 2–10 μl) equivalent of 50 μg of the total protein were then loaded on SDS-PAGE tris-tricine peptide-separating gels. Prestained broad range molecular weight proteins standard (Bio Rad, USA) was used in gel marker lane. Following electrophoresis the gels were electrotransferred onto Immobilon-P polyvinylidene difluoride (PVDF) 0.45 μm membrane (Millipore, USA) in the buffer containing 10 mM 3- [cyclohexyloamino]-1-propanesulfonic acid (CAPS) pH 11, supplemented with 10% methanol. The obtained membrane blots were blocked overnight by gentle agitation in 5% bovine albumin in TST buffer (0.01 M Tris-HCl, pH 7.4, 0.9%NaCl), 0.5% Tween-20). All described procedures were performed at room temperature. Albumin solution was discarded and the membranes were then agitated for 2 hrs in TST with the mouse monoclonal anti-RCAS1 IgM-class antibody, 1: 4 000 dilution (Medical and Biological Laboratories, Japan), polyclonal rabbit IgG antibody against DFF45 (Abcam, UK), monoclonal mouse IgG antibody against beta-Actin (Sigma, USA). The membranes were then subjected to 4 cycles of washings in TST, 5 minutes each, and immersed for agitation in the 1 : 2 000 dilution of SIGMA biotinylated anti-mouse IgM μ-chain specific antibodies for 2 hours. After 4 cycles of washings the membranes were then treated in 1 : 2 000 dilution of ExtrAvidin alkaline phosphatase conjugate (SIGMA, USA) for 2 hours and finally washed twice in TST and twice in TST without Tween-20. Color reaction was developed with the use of Fast Red TR/Naphtol AS-MX tablet set (SIGMA, USA). A sufficient bands intensity was obtained usually following 5-minute period of developing. Obtained immunoblotts were then rinsed with distilled water and air-dried.

#### Computer-aided image analysis

Fluor-S MultiImager (BioRad, USA) was used to scan immunoblotted membranes and a QuantityOne software (BioRad, USA) was used to quantitate band intensities. All calculations were performed on RCAS1 antigen band having molecular mass of 32 kDa, DFF45 protein having molecular mass of 45 kDa and beta-Actin as a 42 kDa band. The intensities of these bands were expressed in arbitrary relative units and one unit (U) was defined as a band intensity produced in the reference sample. This reference sample was randomly chosen but it was exactly the same on all blots and applied always in the same amount. The typical procedure of RCAS1 quantization was as follows: the scanned immunoblot membrane contained one lane of molecular mass standards, while one lane of the sample used as a reference to calibrate RCAS1 amount and 12 lanes containing unknown samples. The location of 32 kDa RCAS1 bands in reference and unknown lanes was identified according to the lane containing molecular mass standard. The 32 kDa RCAS1 band intensities in reference and in unknown lanes were then calculated and expressed in pixel number units. These units were divided by pixel number of reference lane band which resulted in relative intensity units "U". The resulting intensity of reference lane band was always 1.00 U while the intensities of bands from 12 unknown sample lanes on the same membrane changed according to RCAS1 level (e.g., if RCAS1 amount in a given sample was 2 times higher than this in reference sample the result was 2.00 U. If the RCAS1 amount was twice lower, then the result was 0.50 U). As mentioned earlier, because all immunoblots contained the same RCAS1 quantity standard and all lanes were loaded with the same amount of total protein (50 μg), the determined values were highly repetitive and independent of experiment conditions. The RCAS1 total amount in examined tissue samples was recognized relatively, which was necessary because no RCAS1 standard is available. The result always shows relative amount of RCAS1 32 kD antigen in 50 μg of total sample protein. All the bands were analyzed for the recognition of DFF45 and beta-Actin proteins in the same way.

### Immunohistochemistry

The immunohistochemistry method was performed to localize the RCAS1 expression in nasal polyps. The immunohistochemical analysis was performed in the Department of Pathology of the Jagiellonian University. 5 μm thick slides were deparaffinized, rehydrated and rinsed in distilled water. Endogenous peroxidase activity was blocked by 8 min incubation in 3% H2O2 at room temperature. The slides were then rinsed and immersed in boiling citrate buffer (pH 6,0) in a microwave oven with three changes of buffer for 5 minutes each. For the immunolocalisation of RCAS1 the slides were treated with the mouse monoclonal antibody Anti-RCAS1 (Medical and Biological Laboratories, Naka-ku Nagoya, Japan in DAKO Antibody Diluent with Background Reducing Components-DAKO, Denmark, dilution 1:1000) in moist chamber overnight. The slides were subsequently rinsed in TBS buffer (pH 7,6) and incubated with the secondary antibody (DAKO Envision TM+ System Labelled Polymer HRP – anti mouse (DAKO, Denmark) at room temperature for 30 minutes. Visualisation was performed using AEC (3-amino-9-ethyl-carbazole) as a chromogen (AEC Substrate Chromogen ready-to-use, DAKO, Denmark) at room temperature for 10 minutes. Sections were counterstained with hematoxylin and mounted in glycergel. We assumed that positive reactivity for RCAS1 occurred when we observed a granular cytoplasmic (rarely membranous) staining pattern in at least 10% of cells with the following intensity: 0- no reactivity; 1-weak (10–25% positive cells); 2-moderate (26–50% positive cells); 3-strong (51–100% positive cells).

### Statistical analysis

The distribution of variables in the examined groups of patients checked with the use of the Shapiro-Wilk test showed that all of them were different from normal. Therefore, non-parametric testing was employed. Statistical significance between the groups was determined by the Kruskal-Wallis analysis of variance (ANOVA) test. The Mann-Whitney U test was then used when appropriate. Data in Table 2 were presented as means ± standard error of the mean (SEM). A p value of <0.05 was accepted as statistically significant. The Spearman rank test was used to evaluate interclass correlation coefficients. All calculations were carried out with the use of STATISTICA software v. 6 (StatSoft, USA, 2001).

## Authors' contributions

MDW conceived of the study, designed the study, analysed and interpretated data, drafted the manuscript

RT carried out the molecular study

PS participated in the sequence alignment

LW participated in the design of the study, data interpretation and performed the statistical analysis

JS participated in coordination of the study

All authors read and approved the final manuscript.
